# Supplementary material for: Causal Relationship between Aging and Anorexia Nervosa: A White-Matter-Microstructure-Mediated Mendelian Randomization Analysis
Source: Biomedicines. 2024 Aug 16;12(8):1874. doi: 10.3390/biomedicines12081874 (PMC11351342; doi:10.3390/biomedicines12081874)
Supplement: Supplementary file 1 [file biomedicines-12-01874-s001.zip › Supplementary Figure/Figure S4/Singe SNP plot of MR analysis for TL to ubm-b-1554.pdf]

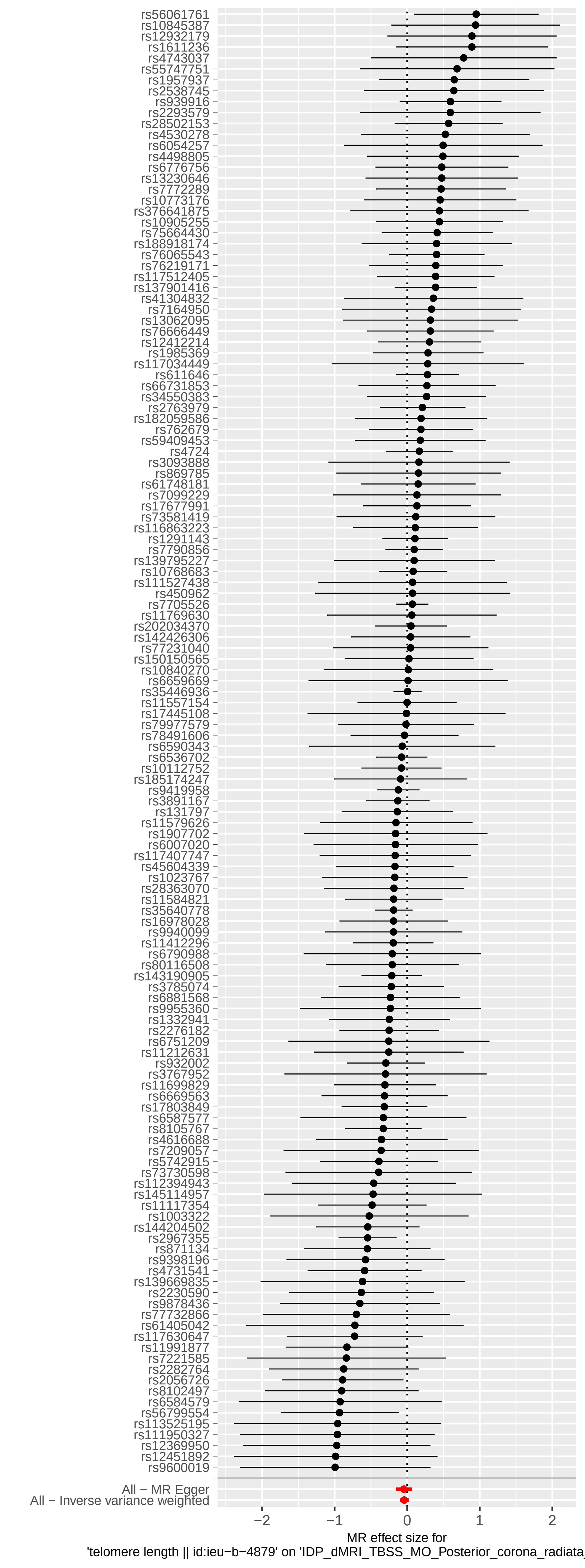

rs56061761
rs10845387
rs12932179
rs1611236
rs4743037
rs55747751
rs1957937
rs2538745
rs939916
rs2293579
rs28502153
rs4530278
rs6054257
rs4498805
rs6776756
rs13230646
rs7772289
rs10773176
rs376641875
rs10905255
rs75664430
rs188918174
rs76065543
rs76219171
rs117512405
rs137901416
rs41304832
rs7164950
rs13062095
rs76666449
rs12412214
rs1985369
rs117034449
rs611646
rs66731853
rs34550383
rs2763979
rs182059586
rs762679
rs59409453
rs4724
rs3093888
rs869785
rs61748181
rs7099229
rs17677991
rs73581419
rs116863223
rs1291143
rs7790856
rs139795227
rs10768683
rs111527438
rs450962
rs7705526
rs11769630
rs202034370
rs142426306
rs77231040
rs150150565
rs10840270
rs6659669
rs35446936
rs11557154
rs17445108
rs79977579
rs78491606
rs6590343
rs6536702
rs10112752
rs185174247
rs9419958
rs3891167
rs131797
rs11579626
rs1907702
rs6007020
rs117407747
rs45604339
rs1023767
rs28363070
rs11584821
rs35640778
rs16978028
rs9940099
rs11412296
rs6790988
rs80116508
rs143190905
rs3785074
rs6881568
rs9955360
rs1332941
rs2276182
rs6751209
rs11212631
rs932002
rs3767952
rs11699829
rs6669563
rs17803849
rs6587577
rs8105767
rs4616688
rs7209057
rs5742915
rs73730598
rs112394943
rs145114957
rs11117354
rs1003322
rs144204502
rs2967355
rs871134
rs9398196
rs4731541
rs139669835
rs2230590
rs9878436
rs77732866
rs61405042
rs117630647
rs11991877
rs7221585
rs2282764
rs2056726
rs8102497
rs6584579
rs56799554
rs113525195
rs111950327
rs12369950
rs12451892
rs9600019
All − MR Egger
All − Inverse variance weighted
−2
−1
0
1
2
MR effect size for
'telomere length || id:ieu−b−4879' on 'IDP_dMRI_TBSS_MO_Posterior_corona_radiata_
